# Hospitals for the Intemperate

**Published:** 1855-01-01

**Authors:** 


### HOSPITALS FOR THE INTEMPERATE.

In one of the early numbers of the *Psychological Journal*, when alluding to the increased prevalence of *Oinomania* the insanity exhibiting itself in a morbidly uncontrollable desire for stimulants, we made, after suggesting the establishment of hospitals for the intemperate, the subjoined remarks :—

"We should like to see this important subject taken up by judicious persons, and much of the fallacy, error, and prejudice in association with it, dispelled. Alas! how many hundreds are annually sacrificed at the shrine of intemperance, whose lives might have been spared had efficient means been resorted to at an early period, before the habit became fixed and confirmed. That there is a disease of the mind manifested solely in an uncontrollable desire for stimulating drinks, we have not a doubt; the more we see of the insane, the stronger is this conviction fastened on our minds. There is ordinary intoxication, and this may, to a certain extent, become a habit; but there is, apart from this, a form of insanity, exhibiting itself almost exclusively in a morbid yearning for intoxicating drinks. We have often seen cases of the kind, and as often have lamented our inability seriously to grapple with them until the disease has extended into positive delirium, and it is only at this stage that the law allows us to interpose."

This suggestion, thrown out by ourselves many years back, has not yet been acted upon in this country. The more the pity. Our transatlantic "go-ahead" friends, who are ever on the alert to meet the pressing exigencies of the time, and are always prominent in every Christian and philanthropic undertaking, are adopting active measures to establish an institution of the kind for the reception and treatment of persons addicted to uncontrollable habits of intemperance, under the designation of

"The Inebriate Asylum."

We call the particular attention of our readers to the following remarks in reference to this noble project. We extract them from the *New York Tribune,* of December 1, 1854.

"We, the undersigned, appointed by the Legislature of the State of New York to organize an institution to be known as the 'United States Inebriate Asylum,' and to act as Commissioners to receive subscriptions to the capital stock of the said Asylum, do herewith submit to the public the following statement:—

"The object of this institution is to provide an asylum for the poor and destitute inebriate, where his physical and moral condition will be alike the care of the physician and the philanthropist, and where his labour may be rendered productive, and of service to his family.

"With the Asylum there will be connected workshops, in which each patient, as soon as his condition will permit, will be employed—thus making the Asylum a self-supporting institution.

"It will be seen that the community will thus be relieved of the burden of maintaining inebriates in alms-houses and prisons, who will be separated from the society of those incarcerated for public crimes, and placed where their inebriety will be treated as a *disease,* and where no efforts will be wanting to produce in them a reformation, and where an income from their labour will be secured to their families, who otherwise would be left to penury and suffering.

"To carry out successfully the great aim of the institution, 50,000 dollars must be raised—this being the amount of capital stock required by the charter. This amount (which can be increased when necessary) is divided into shares of 10 dollars each. Any person wishing to subscribe to the capital stock can send his name, with the amount he will take, to any one of the directors.

"We think it judicious to lease a building or buildings, until suitable edifices can be erected, for the purpose of entering at once, or as soon as practicable, upon the work for which the charter was granted. In accordance with a provision in the act of incorporation, there will be a report on the third Wednesday of January of each year, of the proceedings, expenditures, income, and condition of the Asylum, verified by the affidavit of the President and Treasurer, which report must be filed in the office of the Secretary of State.

"In regard to the necessity of an institution of this character, we cite no less an authority than Dr. Benjamin Rush.

"'To the account of physical remedies,' he says, 'I shall add one more—viz., the establishment of a hospital in every city and town in the United States, for the exclusive reception of hard drinkers. They are as much the objects of public humanity and charity as mad people. They are indeed more hurtful to society than most of the deranged patients of a common hospital would be if they were set at liberty.'

"We are happy in giving assurance that this enterprise meets with the approbation and encouragement of many of the most intelligent and philanthropic members of the community.

"The Directors put forth this brief statement of their object and plan of operations, with the expectation of meeting a quick and cordial response from the benevolent of this and other sections of the country. The call for sympathy and material aid in laying a permanent basis of an institution that promises much for the recovery and salvation of a large number of the human brotherhood, we are confident will not be unheeded.

"This institution is not designed to conflict with any other methods for saving the inebriate. There is nothing, we believe, similar to it in this or any other country.

"Thousands will look to it for help, and help they should and must have.

"That which was worth creating is worth preserving. The Benevolent Father puts it within our power to save those who are ready to perish. To rescue a fellow-being from physical and spiritual thraldom is worthy of the exercise of the highest talents and the purest love. To redeem from ruin is greater than to create. To turn from vice to purity, from darkness to light, from death to life; to make him possessed of a free, enlarged, and beautified existence, is a divine mission.

"Everywhere goes up the wail of wrecked humanity, of prostrate and suffering brothers; from every side comes the cry for help. They are the true workers who respond to this cry. They are enriched by giving, and blessed in blessing.

"Fellow-citizens, fathers, brothers, and sisters! give us your aid in this branch of beneficence, and the blessing of multitudes will be your reward."

This subject is ably handled in a recent report of the trustees, and of the Butler Hospital for the Insane (U.S. America), in which the form of insanity exhibiting itself in an involuntary and irresistible propensity to drink is fully and accurately described. The difficulties that beset the physician in the treatment of these cases are fairly represented by the writer:—On complying with certain (legal) conditions, we are authorized to hold in confinement persons who are insane; but no law of the land would justify us in depriving men of their liberty for any other cause, however commendable the object. Now, the class of persons in question, while in the paroxysm, or suffering under its immediate effects, may, in any proper sense of the term, be called insane, and so long we have an unquestionable right to hold them. When, however, this condition passes away, as it usually does within a few days or weeks, and the mind resumes its perfect consciousness, what are we to do? The person claims his liberty, while nobody doubts that he would use it only to advance another step in the road to bodily and mental ruin. Here seems to be a conflict of duties, and with every disposition to do right, I do not see how we can help compromising either the happiness of families or the rights of individuals. The friends are desirous of giving the person the benefit of the only measure which promises relief—a protracted abstinence from intoxicating drinks. The measure is prompted by kindness and duty, but nothing short of confinement will ensure its accomplishment, and they appeal to us to aid them in their laudable design with the means and appliances at our command. Prompted by similar motives, we consent to receive the person and give him the benefit of the peculiar discipline and management of a hospital for the insane. In most cases we encounter no opposition. The person may protest against the measure which deprives him of liberty, but he fails to obtain any support or countenance. The right of friends or guardians to subject him to any reasonable management which promises to restore his appetites and passions to a healthy condition, remains unquestioned, and we are as little disturbed by meddlesome interference as in any other class of cases. But it occasionally happens that the person invokes the aid of those who disregard altogether the moral aspects of the case, and we are threatened with the terrors of the law, for holding in confinement men who are neither insane nor guilty of crime. This is an embarrassing position. On the one hand we are prompted by a sense of right and duty to avoid all doubtful constructions of the law; on the other, our sympathies are excited by the agony of friends whose hearts have been torn by repeated outrages upon public order and the peace of the domestic circle; whose strongest efforts have been needed to avert painful exposures, if not the most disgraceful penalties of the law; and who dread the renewal of those scenes in which every sentiment of delicacy, honour, propriety, and right seemed to have given place to a savage, grovelling fury. Whatever course we take—whether we shun collision with the law by yielding at once to its demands, or, strong in the rectitude of our intentions and objects, pursue the even tenor of our way and abide the consequences—we assume a responsibility which ought not, in justice, to be imposed upon us. It would seem, then, to be a very proper conclusion, that if we are expected to receive the class of persons in question, we must be invested with the requisite legal authority. Let the legislature enact that habitual drunkenness shall be subjected to all the disabilities of insanity, and then we may engage in a work of humanity without infringing upon the right of individuals."

---

